# Cell-free protein synthesis and vesicle systems for programmable therapeutic manufacturing and delivery

**DOI:** 10.1186/s13036-025-00523-x

**Published:** 2025-06-05

**Authors:** Wonhee Kim, Jinjoo Han, Shraddha Chauhan, Jeong Wook Lee

**Affiliations:** 1 Division of Interdisciplinary Bioscience and Bioengineering, Pohang University of Science and Technology (POSTECH), Pohang, Gyeongbuk 37673 Korea; 2 Department of Chemical Engineering, Pohang University of Science and Technology (POSTECH), Pohang, Gyeongbuk 37673 Korea; 3 Graduate School of Convergence Science and Technology, Pohang University of Science and Technology (POSTECH), Pohang, Gyeongbuk 37673 Korea; 4 School of Integrated Technology, Yonsei University (POSTECH-Yonsei Open Campus), Pohang, Gyeongbuk 37673 Korea

**Keywords:** Cell-free protein synthesis system, Therapeutic proteins, Genetic circuits, Vesicles, Drug delivery

## Abstract

The convergence of cell-free protein synthesis (CFPS) and vesicle-based delivery platforms presents a promising avenue for therapeutic development. The open environment of CFPS offers precise control over protein synthesis by enabling the modulation of synthetic conditions. Additionally, vesicle-based platforms provide enhanced stability, bioavailability, and targeted delivery. This synergy facilitates the efficient production of complex proteins—including membrane proteins, antibody fragments, and proteins requiring post-translational modifications (PTMs)—and supports novel drug delivery strategies. While existing reviews have covered synthetic cells and biomanufacturing broadly, a dedicated analysis of CFPS system-containing vesicles (CFVs) for therapeutic applications remains absent from the literature. This review addresses this knowledge gap by providing a comprehensive examination of CFVs, highlighting their potential as programmable drug delivery platforms through the integration of genetic circuits. It emphasizes the advantages of CFPS over traditional cell-based approaches and explores the synergistic benefits of combining CFPS with various vesicle systems. These systems offer dynamic control over therapeutic protein production and targeted delivery, enabling precise responses to specific signals in complex environments. Although challenges such as low protein yield and imperfect targeting remain, potential optimization strategies are discussed. This analysis highlights the significant potential of integrating CFPS and vesicle-based delivery to advance biomanufacturing, therapeutic development, and synthetic cell systems, thereby opening new avenues in medicine and healthcare.

## Introduction

The development of therapeutics requires innovative techniques that allow flexible, accurate, and efficient drug synthesis. Advances in synthetic biology have enabled researchers to manipulate living cells to produce therapeutics, focusing not only on manufacturing medicines but also on optimizing their biological functions [1]. Traditional cell-based systems harness cellular machinery to provide a natural environment for protein synthesis. However, these approaches are often time-consuming, prone to contamination, challenging to scale, and unsuitable for producing membrane-bound or toxic proteins [2]. In contrast, cell-free protein synthesis (CFPS), which occurs in vitro*,* provides an open reaction environment that allows direct control over protein synthesis [3]. Unlike conventional cell-based methods, CFPS avoids challenges such as cytotoxicity caused by excessive protein expression, thereby enabling more efficient protein production [4]. Moreover, the ability to manipulate the protein expression environment facilitates the optimization of conditions for the production of proteins with specific properties. This not only improves the protein expression level but also increases its activity.

Recently, the biosynthetic potential of CFPS has gained increasing recognition in the field of biotherapeutic development [2]. CFPS enables the production of complex therapeutics—such as membrane proteins and proteins requiring post-translational modifications (PTMs)—that are difficult to manufacture on a large scale because of their intricate biological functions. Numerous studies have demonstrated the efficient synthesis of various therapeutic molecules, including receptors, antibodies, and vaccine antigens, highlighting CFPS as a promising platform for biotherapeutic applications. Similarly, vesicle-based delivery systems such as liposomes, polymersomes, and microsomes can effectively transport therapeutics. These vesicles offer various advantages, including enhanced stability, improved bioavailability, and the ability to precisely target therapeutic agents to specific locations within the body [5, 6]. The integration of CFPS with vesicle-based drug delivery systems enables precise protein production, safe drug encapsulation, controlled drug release, and targeted delivery [5]. This powerful synergy simultaneously streamlines biotherapeutic production and enhances therapeutic outcomes through improved protein stability, increased bioavailability, and precise spatiotemporal control of drug delivery.

Despite its potential, CFVs-based biotherapeutics face challenges, including higher protein yields, advanced gene regulation strategies, decreased immunogenicity, and improved vesicle targeting efficiency.

This review explores the integration of CFPS and vesicle-based delivery, highlighting its advantages over conventional cell-based technologies from a biomanufacturing perspective. CFPS-embedded vesicles have various applications ranging from protein production to synthetic cell generation. A detailed analysis of the advantages and limitations of CFVs based on vesicle type is provided, along with a discussion of synergistic approaches, such as therapeutic delivery, controlled release mechanisms, and programmed therapeutic expression.

## Cell-free protein synthesis systems and vesicles

### CFPS systems

**Fig. 1:**
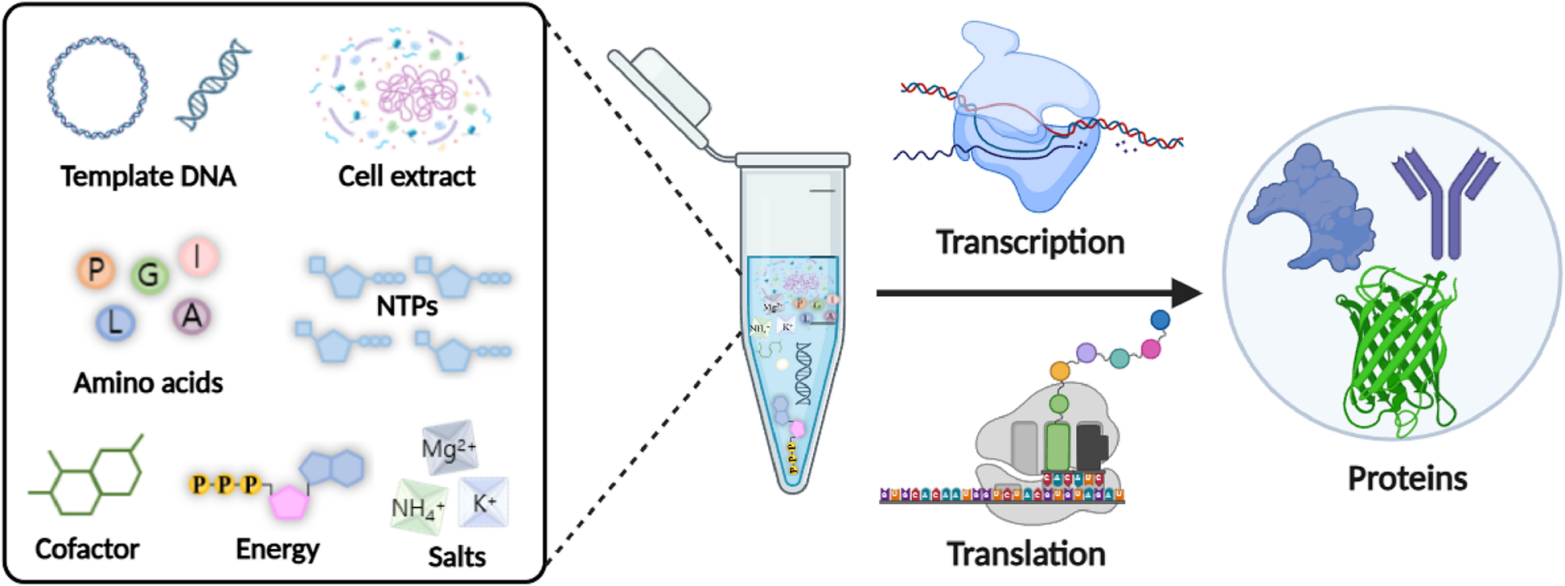
Schematic representation of CFPS: In a test tube, the components of a CFPS reaction are mixed. This mixture comprises the molecular machinery of the cellular lysate as well as DNA, amino acids, and energy buffers. This combination facilitates the production of functional proteins through transcription and translation processes

CFPS systems have garnered considerable attention because of their unique advantages in basic science and biotechnology [7, 8]. Unlike traditional cell-based biomanufacturing, which has limitations in terms of scalability, protein complexity, and cost, CFPS systems facilitate rapid protein production in vitro (Fig. 1). By using cellular machinery in a controlled environment, CFPS eliminates time-consuming cell culture steps, allowing high-throughput protein synthesis [9]. For example, extract-based CFPS systems can efficiently express 24 open reading frames in a single day, which is significantly faster than conventional cellular expression methods [10].

In living cells, excessive protein production can lead to cytotoxicity and inclusion body formation owing to host cell stress resulting from resource depletion. Moreover, excessive protein production within cells can disrupt proper protein folding and quality control, thereby impairing cell viability and function. These limitations underscore CFPS as an efficient and scalable alternative to traditional in vivo expression methods. The absence of cellular constraints in CFPS mitigates the trade-off between protein production and cell growth [7, 11]. Furthermore, CFPS offers real-time control over key parameters such as pH, temperature, and substrate concentration, thereby optimizing protein expression more efficiently than cell-based methods [12, 13]. Adjusting reaction buffer concentrations in a cell-free system accelerates optimization and helps identify important parameters [14]. Additionally, researchers can simplify the characterization process and adjust the reaction environment by introducing necessary cofactors, chaperones, and enzymes [15, 16]. These properties are particularly useful for dealing with complex proteins that may be unstable or misfolded. For example, Focke et al*.* demonstrated the correct folding of membrane proteins, including the K^+^ channel KcsA, the voltage-gated K^+^ channel MVP, and the amino acid transporter LeuT, using CFPS [17]. The inherent flexibility of CFPS allows precise manipulation and optimization of reaction conditions, thereby facilitating high protein yields.

Notably, the streamlined process in a cell-free environment minimizes complex purification steps, thereby increasing the overall efficiency of the synthesis process [18]. By leveraging CFPS, researchers can efficiently produce difficult-to-express proteins, leading to a better understanding of their previously unknown protein characteristics, functions, and interactions with other molecules [15]. Collectively, the versatility and efficiency of CFPS make it a valuable tool in synthetic biology, offering a novel approach for protein synthesis with enhanced control and productivity.

### Advantages of CFPS systems in the biomanufacturing of complex therapeutics

Biotherapeutics, including mRNA-, peptide-, and protein-based treatments, are derived from naturally occurring biomolecules, allowing them to target specific cells or tissues with greater precision and fewer side effects [19]. Many biotherapeutics require complex PTMs, such as glycosylation and disulfide bond formation, which are enzymatically catalyzed by specific enzymes like glycosyltransferases and protein disulfide isomerases. However, achieving proper folding in large-scale cellular expression can be challenging. CFPS provides a controlled alternative to traditional cell-based expression methods, enabling more efficient regulation of complex protein modifications while avoiding cell growth constraints and metabolic stress [7, 20]. Compared to conventional cell-based methods, CFPS systems offer significant advantages in terms of protein synthesis efficiency (Table 1).

**Table 1:** Comparative analysis of traditional cell-based and CFPS systems for biomanufacturing

Feature	Traditional Cell-Based Biomanufacturing	Cell-Free Protein Synthesis (CFPS)	Ref
Process	Days to weeks	Minutes to hours	[21]
Scalability	Limited by cell growth and culture capacity	Highly scalable and adaptable to demand	[21]
Post-translational modifications (PTMs)	Limited options depending on host cell	Wider range of PTMs achievable with specific systems	[21]
Protein complexity	Limited to proteins compatible with cellular expression	Can handle complex, toxic, or membrane proteins	[22]
Setup	Requires extensive equipment and cell culture facilities	Simpler setup, potentially portable	[23]
Applications	Wide range, but limited by cellular constraints	High-throughput screening	[11, 24]

Although CFPS has successfully facilitated the folding of simple proteins, producing complex therapeutics containing disulfide bonds remains challenging. However, a key advancement in this area involves the discovery of DsbC, an enzyme that can catalyze disulfide bond exchange in *E. coli*. Goerke et al. demonstrated that pretreating cell extracts with iodoacetamide (IAM) inactivated cytosolic redox enzymes, stabilizing the redox potential. Additionally, a glutathione buffer consisting of oxidized glutathione and reduced glutathione facilitated disulfide bond formation and exchange via DsbC. They showed that using various cell extracts enabled the large-scale production of proteins containing up to 24 disulfide bonds, ranging in size from 14.3–53.2 kDa. Furthermore, optimizing IAM concentration and available membrane vesicle surface area enhanced disulfide bond formation [20].

Additionally, the endotoxin-free *E. coli*-based CFPS system has attracted attention for its rapid therapeutic protein production [25]. However, bacterial CFPS systems inherently lack the cellular machinery required for complex PTMs, which limits their broader application. To address this, researchers have developed prokaryotic-based CFPS systems capable of performing PTMs, such as acetylation, glycosylation, phosphorylation, and methylation, which has substantially expanded their utility in protein therapeutics. For example, Jaroentomeechai et al. engineered a glyco-optimized *E. coli* strain to achieve glycosylation. This was accomplished by selectively enriching cell extracts with glycosylation components, including oligosaccharide transferases and lipid-linked oligosaccharides [26]. Using an *E. coli*-based CFPS system, researchers have successfully demonstrated efficient site-specific glycosylation of target proteins in a one-pot reaction system [27, 28]. This highlights the versatility and capabilities of prokaryote-based CFPS systems and represents a significant step forward in protein synthesis and PTMs. These advances have increased the commercial utility of CFPS systems and supported rapid and efficient product and process development.

### Synergistic effects of integrating CFPS systems and vesicles in biotherapeutics production

**Fig. 2:**
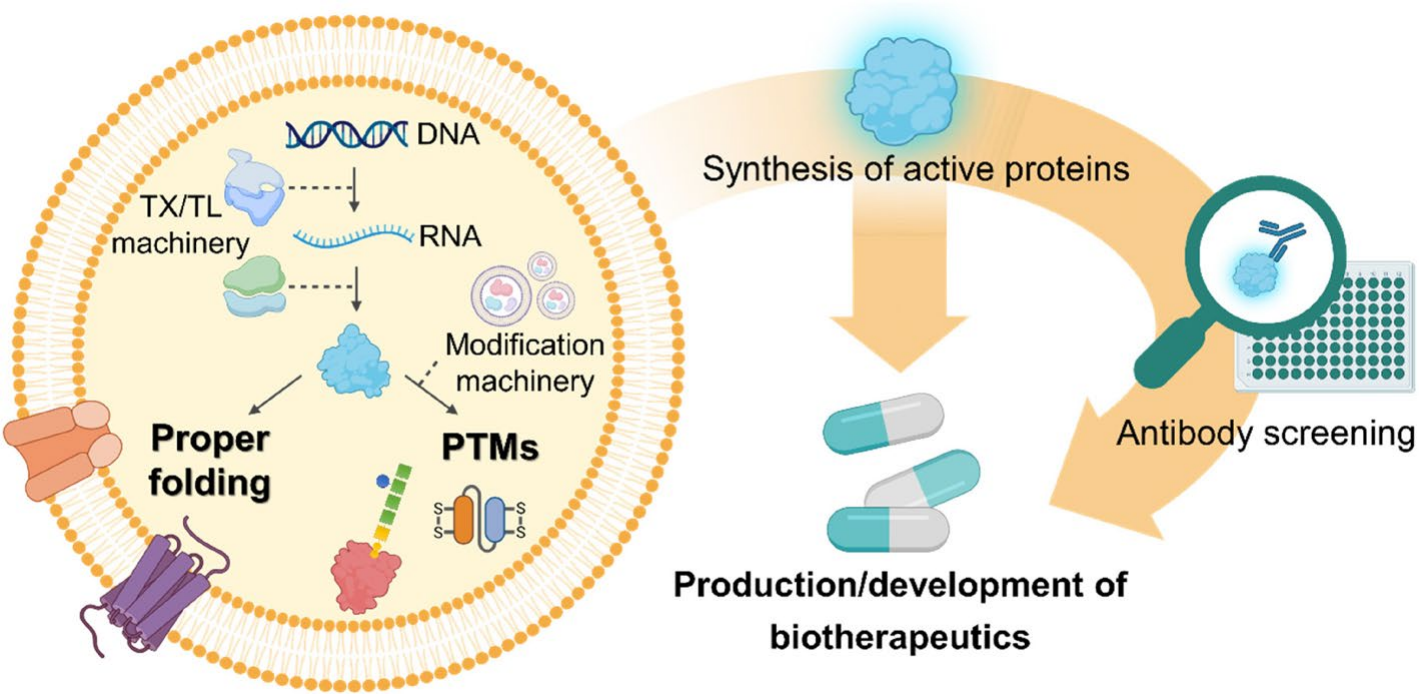
Schematic representation of a vesicle-assisted CFPS system for biotherapeutic production. The vesicle either encapsulates the transcription/translation (TX/TL) machinery or provides protein modification components, facilitating proper folding and PTMs. This synergistic integration of vesicle and CFPS systems enables the efficient synthesis of active, functional proteins, thereby accelerating the development of biotherapeutics

Membrane protein research is important for maximizing the efficacy of biotherapeutics because it directly affects their activity and function. Integrating CFPS with vesicle technologies enables the production of membrane proteins in a lipid bilayer environment similar to physiological conditions, improving their stability and functionality. Vesicles facilitate the study of membrane protein properties by mimicking the natural cell membrane structure (Fig. 2), whereas CFPS systems allow precise regulation of protein folding and synthesis modifying reaction conditions [29]. These controlled manipulations make in vitro systems valuable for exploring protein behavior and function [30, 31]. For example, G protein-coupled receptors (GPCRs) are important drug targets; however, they are difficult to overexpress in cellular systems and existing screening methods can lead to the denaturation of GPCRs. To address this, Takeda et al. efficiently synthesized 25 different GPCRs using a wheat germ-based CFPS system, stabilizing them with liposomes to prevent denaturation. They utilized a biotinylated liposome-based interaction assay to confirm GPCR-antibody interactions, enabling cost-effective and efficient antibody screening [32]. Similarly, recombinant membrane proteins, such as outer membrane porin F, which are challenging to overexpress in living cells, have been successfully produced using CFPS with rapid screening of various liposome compositions [33]. Membrane proteins such as bacteriorhodopsin and GPCRs are key drug targets. CFPS facilitates their correct insertion into vesicle membranes, enabling direct functional assays in synthetic lipid bilayers [32, 34–36]. Additionally, vesicles serve as nanocarriers for biotherapeutic delivery, offering a platform for the design, characterization, and development of safe and effective drug transport systems [37–39].

Furthermore, eukaryotic-based CFPS systems containing endoplasmic reticulum (ER)-derived vesicles have the potential for a wide range of biotechnological applications. Notably, these systems enable PTMs, which are crucial for the functionality of proteins, such as receptors, hormones, and antibodies. For example, an insect cell-based CFPS system facilitates the synthesis, transport, and accumulation of single-chain variable fragments (scFvs) within microsomal structures [40]. The proper oxidative folding of scFvs via disulfide bond formation is crucial for their stability and therapeutic efficacy. By using ER-containing insect cell lysates with active microsomal structures, researchers successfully translocated scFVs into ER-derived vesicles, where they underwent PTMs. Additionally, Hershewe et al. engineered an *E. coli*-based CFPS system to perform glycosylation by enriching vesicles with heterologous membrane-bound glycosylation machinery [26, 41]. This strategy enabled the synthesis of diverse glycoproteins, including model and human glycoproteins, and demonstrated the potential of CFPS systems as versatile platforms for producing protective conjugate vaccines. These advances demonstrate how CFPS systems with PTM capabilities can expand the spectrum of expressible proteins, thereby enhancing their therapeutic properties for biotechnological applications.

### Types of vesicles for CFPS systems

Various vesicle types have been employed in CFPS systems for applications such as microreactor or artificial cell formation, membrane protein synthesis, and functionalization. Vesicles play two important roles: 1) enclosing CFPS systems to create compartmentalized environments for biological reactions; 2) acting as scaffolds for membrane protein integration. CFVs can be customized based on vesicle type, size, method of modification, and the organism from which the CFPS system is derived. Notably, these systems facilitate gene expression across various host cells, ranging from bacteria such as *E. coli* to mammalian cells. This makes them powerful tools for biomimetic and biotechnological applications when combined with the functional complexity of artificial cells. In this section, studies that have utilized vesicles for CFPS are reviewed, and the advantages and disadvantages of various vesicle types for CFPS systems are discussed (Table 2).

**Table 2:** Comparison of vesicle types for CFPS

Vesicle	Diameter	Membrane thickness	Cell-free system	Goal	Main work	Product	Pros	Cons
Liposome	0.02–100 um	3–5 nm	*E. coli* [34, 35, 42–48] PURE [49–61] WGE [32, 36–38] RRL [39]	Bottom-up synthesis of an artificial cell	Fabrication of monodisperse liposomes	GFP [42, 46, 47, 49, 52, 53, 58, 59] mCherry [50]	- High encapsulation capacity - Most closely resembles the biological cell - Biocompatibility and biodegradability - Incorporation of membrane protein - Technical simplicity - Able to control structural characteristics - High scalability - Mature studies	- Relatively less uniformity of diameter - Low stability - Low chemical versatility
					Expression of membrane proteins	α-hemolysin [43] 85 membrane proteins [54] gp91^phox^ [62]		
					Establishment of replication system	Qβ replicase [44, 51, 57] β-gluocoronidase [51]		
					Exploring ability to communicate	Quorum molecules [45] α-hemolysin [43, 63]		
				Manufacturing products valuable for industry or research	Expression, screening, characterization, and engineering of proteins	Sphingomyelin synthase [37, 38] Stearoyl-CoA desaturase [36] Microtubule [56] GPAT [55] LPAAT [55]		
					Production and analysis of drug target protein	Bacteriorhodpsin [34] GPCR [32, 35]		
					Formation of proteoliposome	Connexin 43 [39] αHER2 affibody [60] scFv [60] NarX-L [48]		
					Improving expression of membrane protein	MscL [61]		
Polymersome	0.2–150 um	5–50 nm	*E. coli* [64–67] PURE [30] WGE [68, 69]	Bottom-up synthesis of an artificial cell	Fabrication of monodisperse polymersomes	MreB-RFP [64] mCherry [66]	- Similar production method to that of liposome - Mechanically and chemically stable - Incorporation of membrane proteins - Easy modification - Potential for functionalization	- Less flexibility and permeability - Little biocompatibility - Low encapsulation efficiency - Low scalability
					Polymersome formation based on ELP	GFP [67] ELP [65]		
				Manufacturing products valuable for industry or research	Production and analysis of drug target protein	MscL-GFP [30] GPCR [68]		
					Formation of proteopolymersome	Cldn2 [69]		
Microsome	0.02–0.2 um	3–4.5 nm	WGE [70] RRL [70] *Sf*21 [40, 71–77] K562 [76] CHO [76, 78–80]	Manufacturing products valuable for industry or research	Post-translational modification of target protein or therapeutics	scFv [40, 71–73] EGFR [73] EPO [78] IgG [80]	- Efficient incorporation of membrane protein - Proper post-translational modification of target protein - Does not require detergent solubilization step	- Higher complexity of CFPS system - Sophisticated composition may reduce translation efficiency - Cannot be directly modified - Low scalability
					Expression and analysis of membrane protein	Preprolactin [70] EGFR [76, 77] KcsA [74, 80] GPCR [75]		
				Improving eukaryotic CFPS		EGFR [76, 77]		

#### Liposomes

Liposomes are microscopic sphere-shaped structures composed of lipid bilayers. They are categorized based on several factors, including their size (small, large, or giant), number of lipid layers (single, few, or many), lipid composition, and electric charge (neutral, negatively, or positively charged) [81]. Unilamellar liposomes, which are characterized by a single lipid bilayer, are monodisperse, making them ideal for quantitative analysis in laboratory settings. This makes them ideal for widespread use in CFPS systems to study biochemical reactions [34, 42, 49, 82–85]. In CFPS, liposomes function as microreactors and scaffolds for membrane protein production, as described in the previous section.

Integrating CFPS with liposomes offers several advantages over other vesicle types. First, liposomes closely resemble natural biological cells in terms of membrane components, structure, and size, making them effective artificial cell models when encapsulated in CFPS systems. They are highly biocompatible and biodegradable, and minimize immune responses, making them suitable therapeutic delivery vehicles [86, 87]. Additionally, liposomes exhibit favorable scalability and reproducibility, with established industrial-scale production methods including microfluidic systems and ethanol injection techniques [88–90]. Furthermore, liposomes produced through water–oil emulsion and thin-film hydration technologies show high encapsulation efficiency, with the water–oil emulsion method resulting in an entrapment efficiency of over 80% [91–93]. Given the high costs associated with extract- and enzyme-based CFPS systems, maximizing encapsulation efficiency is important for optimizing process efficiency and cost-effectiveness. Finally, liposomes can be fine-tuned for endogenous expression, membrane protein integration, and various structural properties, making them easy to modify for various research and application requirements.

Using synthetic cell populations, Gonzales et al. quantified gene expression dynamics in individual liposomes using computational modeling, establishing a statistically robust methodology for analyzing CFPS systems [50]. Numerous studies have focused on imparting native cellular properties to these liposomal systems. For example, the expression of α-hemolysin nanopores within the lipid membrane enables molecular transport across the liposomal bilayer. These nanopores prolong the duration of CFPS and increase protein yield by preventing nutrient depletion during synthesis.

Another example of integrating CFPS with liposomes is a simplified RNA replication system that employs Qβ replicase, an RNA-dependent RNA polymerase. This system was designed so that an RNA template could serve as a replication template and a source of replicase. Encapsulation within liposomes allowed the evaluation of the effects of liposome size on replication activity. The results revealed that smaller liposomes exhibited increased RNA replication [44, 51].

Liposomes capable of interacting with natural cells have also been developed [45, 52, 63]. For example, liposomes encapsulating isopropyl β-D-1-thiogalactopyranoside (IPTG) and a theophylline-sensing genetic device encoding α-hemolysin can induce gene expression in the presence of theophylline by delivering IPTG to bacterial cells through the lipid membrane [63]. Another study introduced genetic constructs encoding quorum signaling molecules into the liposomes. These liposomes sense or synthesize quorum-signaling molecules secreted by bacteria, thereby facilitating interactions with natural cells [52].

Although liposomes are useful in CFPS research, they have several limitations. Their small size and lack of uniformity can impact the volume and surface-area-to-volume ratio of encapsulated CFPS systems, leading to variations in gene expression and membrane protein concentration [94]. Additionally, multilamellar liposomes exhibit heterogenous internal structures and encapsulated component concentrations, complicating in vitro transcription/translation analysis and CFPS integration [53]. Moreover, liposomes are susceptible to heat, physical forces, detergents, and environmental changes, including temperature, light, and pH variations, which can compromise their integrity [95]. Although membrane stiffness increases with cholesterol, minor fluctuations during CFPS responses can cause instability. When used as a therapeutic delivery system, the limited chemical diversity of liposomes must also be considered.

#### Polymersome

Polymersomes are bilayer membrane structures composed of synthetic amphiphilic polymers and have garnered attention as CFPS-compatible vesicles. They consist of block copolymers such as poly(ethylene glycol)-block-poly(lactic acid), poly(ethylene oxide)-block-poly(butadiene), and elastin-like polypeptide (ELP), with sizes ranging from 100 nm to 150 μm [96]. Their high modularity allows for adjustable toxicity, stability, and biocompatibility, making them well-suited for drug delivery systems. Recent advancements have enabled their use in cancer diagnosis and treatment through the encapsulation of diagnostic and therapeutic agents. Additionally, polymersomes have fewer side effects owing to their biocompatibility and biodegradability.

Efforts have been made to integrate polymersomes with CFPS systems as novel approaches to synthetic cell development. Martino et al. developed a microfluidic capillary device to generate polymersomes that effectively mimic real cell populations by encapsulating CFPS systems with high-size homogeneity. Vogele et al. fabricated polymersomes and demonstrated their growth potential [64, 65]. Additionally, polymersomes have enabled endogenous expression and integration of active membrane-associated proteins, facilitating the rapid and efficient expression of proteins such as MreB [64]. Notably, GPCRs have been embedded in polymersome membranes for ligand-binding analysis. De Hoog et al. measured the binding dynamics between polymersome-assisted expressed GPCRs and their antibodies [68, 97]. These results demonstrate the suitability of polymersomes coupled with CFPS as a platform for the production and analysis of membrane proteins that are difficult to express and characterize because of their inherent instability outside the membrane.

Furthermore, a key advantage of polymersomes is their exceptional robustness. By adjusting the chemical composition of the block copolymer, the physical properties of the membrane, such as thickness, elasticity, and permeability, can be controlled, resulting in better mechanical stability than liposomes [98]. These properties can be tailored to specific molecular weights and compositions, enabling the creation of highly functional synthetic vesicles, including osmotic-responsive vesicles. Additionally, CFPS expands polymersome diversity by incorporating transmembrane proteins and enzymes, thereby enhancing their functionality. Moreover, they offer high loading capacity for both vesicular and hydrophilic motifs, making them suitable for minimizing the side effects associated with drug delivery [99]. However, block copolymer membranes are thicker and larger than phospholipid-based membranes. The thicker the membrane, the greater the strength; however, the hydrophobic blocks may become entangled with each other, reducing flexibility and permeability [100]. These properties limit their biocompatibility and material transport applications. Furthermore, conventional polymersome formation methods face challenges associated with size uniformity and encapsulation efficiency. Although microfluidic technology has been introduced to address these issues, achieving perfect uniformity remains challenging. While scalable techniques such as microfluidics and flash nanoprecipitation have shown promise in some studies, the overall scalability of polymersome production is still under debate [101, 102].

#### Microsomes

Microsomes are endogenous vesicles derived from the ER. They are formed during cell lysis and have a double-layer membrane structure with a size of 20–200 nm. The membrane composition and thickness of the ER-derived microsomes closely resemble those of the ER. Eukaryote-based CFPS systems primarily use ER extracts from insect cells or Chinese hamster ovary (CHO) cells, which naturally contain microsomes or can be supplemented with purified microsomes. Recently, cell-free methods have proven suitable for synthesizing scFvs, the smallest recombinant antibody format containing the entire antigen-binding site. The CFPS platform successfully formed functional scFvs with disulfide bonds; scFv candidates were screened, and signal peptide efficiency was investigated [40, 71, 72]. Additionally, microsomes in the CFPS system facilitate the expression of functional membrane proteins and protein glycosylation [73–75].

The main advantage of microsomes is their ability to support PTMs such as disulfide bond formation, glycosylation, phosphorylation, and lipid modifications. This capability mimics the key PTM processes occurring in the ER of cells, promoting proper protein folding and function. Additionally, microsomes contain ER translocation machinery, which helps proteins move efficiently into the membrane via signal peptides [25, 78, 103, 104]. After CFPS, the integrated proteins can be easily purified without tags, detergents, or laborious isolation procedures, enhancing experimental efficiency and simplifying protein analysis.

However, incorporating microsomes into CFPS increases system complexity, making it challenging to establish the optimal conditions for protein synthesis. Complex cell extracts, often regarded as “black boxes,” pose challenges in comprehending and characterizing the native machinery within them. The complexity of the microsome-derived elements may weaken the regulation of biological reactions associated with protein expression. Additionally, microsomal modifications such as cholesterol insertion or phospholipid PEGylation are difficult to achieve, which may lead to limitations in advanced CFPS engineering. Furthermore, their scalability is inherently constrained by reliance on biological sources, low production yield, and the labor-intensive nature of ultracentrifugation-based preparation [105]. These limitations highlight the need for further improvements to enhance their practicality and broaden their applicability in CFPS engineering.

## Exploring CFPS system-containing vesicles for therapeutic delivery

**Fig. 3:**
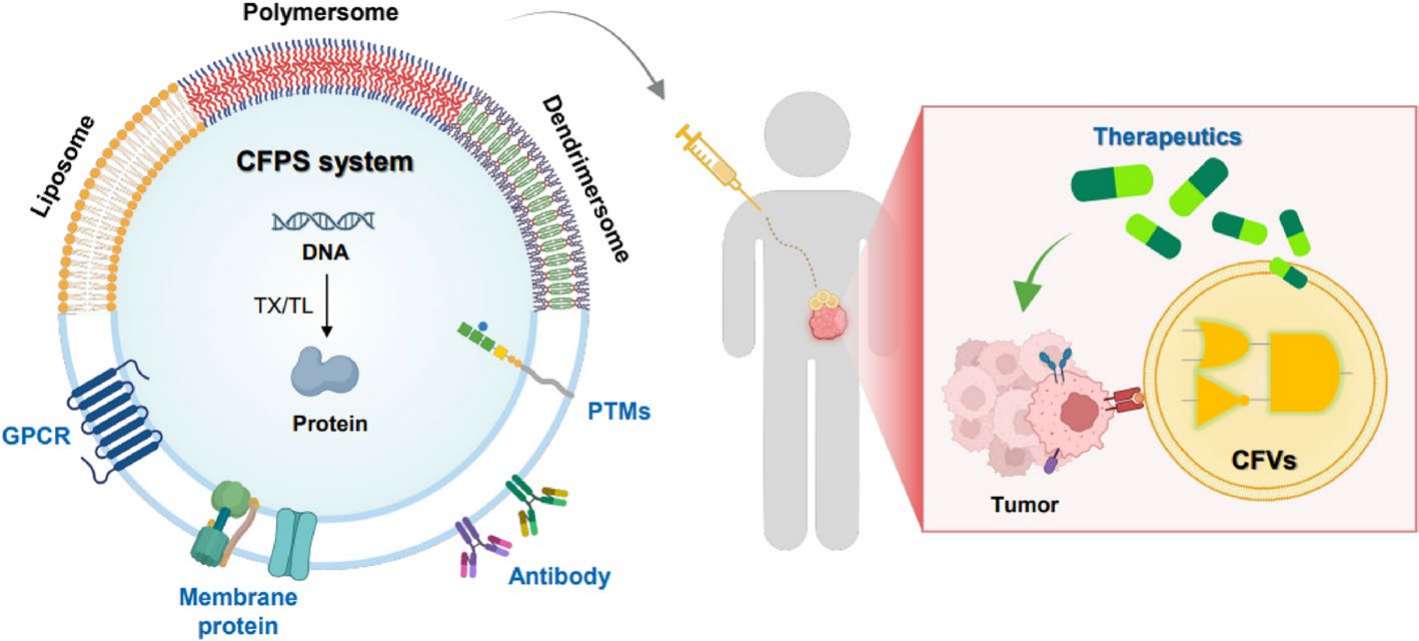
Schematic illustration of a CFV-based drug delivery system integrating CFPS and programmable genetic circuits. CFVs contain modular genetic components that enable controlled production of therapeutic proteins

In conventional drug delivery, vesicles function as carriers that transport and release cargo at target sites. In contrast, CFVs go beyond simple carriers and act as mobile “factories” capable of synthesizing therapeutic molecules on demand (Fig. 3). Therefore, CFV systems offer numerous advantages and overcome the critical limitations of existing drug delivery systems. This section provides a novel perspective on CFVs as distinct drug delivery systems.

### Beyond controlled release: programmed manufacturing of therapeutics

**Fig. 4:**
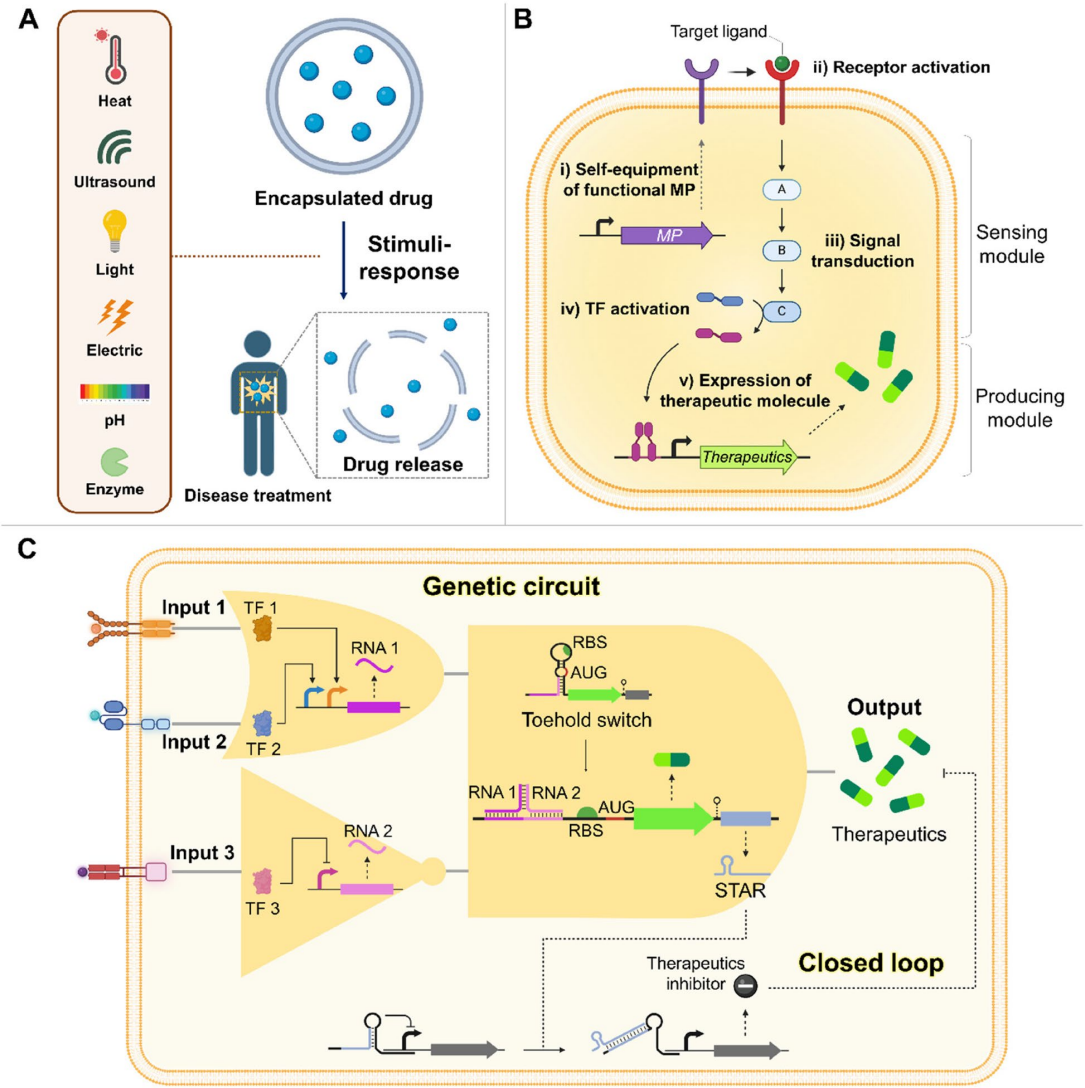
Regulation of the release or expression of therapeutic molecules. **A** Conventional controlled release systems. Drug release is triggered by external stimuli such as heat, ultrasound, light, or electrical signals, as well as by changes in pH or the action of specific enzymes on the drug delivery vehicle. **B** Programmed expression of therapeutic molecules through CFVs. It involves two key components: a sensing module to recognize target molecules or the environment and a production module to generate therapeutic molecules. **C** More sophisticated regulation of therapeutic molecules via synthetic genetic circuits in CFVs. An example of precise drug production regulated by a genetic circuit that manages multiple inputs and employs negative feedback is provided. MP, membrane protein; TF, transcription factor; RBS, ribosome binding site; AUG, start codon; STAR, small transcription-activating RNA

Classical drug delivery systems such as hydrogels, polymeric nanoparticles, micelles, liposomes, and lipid nanoparticles rely primarily on passive uptake and accumulation at target sites. In contrast, controlled drug delivery systems allow the precise regulation of the timing and location of drug release [106, 107]. This control is clinically advantageous because it maintains a constant drug concentration in the blood, provides long-term therapeutic effects, and reduces dosing frequency [108]. Recent developments in drug delivery technologies have focused on achieving controlled drug release. Many delivery systems have been designed to integrate biochemical elements and stimulus–response mechanisms to release the drug in situ after reaching the target site (Fig. 4A). These stimuli may be induced by external factors (e.g., heat, light, and ultrasound) or respond to physiological changes (e.g., pH shifts or biomolecular fluctuations). However, nonspecific therapeutic delivery and undue accumulation remain challenging, suggesting the need for improved strategies to control drug release more precisely and effectively [108, 109].

CFVs address these limitations by releasing drugs upon stimulation and synthesizing therapeutic molecules at specific times and locations. This is achieved by integrating a genetic cassette containing a detection module and a therapeutic production module, allowing precise control over expression and function (Fig. 4B). For example, CFVs can express receptors on their surfaces that detect cancer-related biomarkers or the tumor microenvironment. Ligand recognition activates receptor-mediated signaling pathways, thereby activating transcription factors and resulting in the expression of therapeutic molecules. Peruzzi et al*.* recently demonstrated active signal transduction in a CFPS system using the bacterial two-component system, NarX-NarL [48]. In this system, liposome-synthesized NarX detected nitrate and activated the transcription factor NarL, which induced reporter gene expression. As another example, Adamala et al. showed that liposome encapsulation combined with CFPS systems enables modularization and controlled compartmentalization of genetic circuits and cascades. This approach facilitated the construction of combinatorial genetic circuits within liposomes, allowing precise regulation of gene expression and function in response to external signals and inter-liposomal communication [110]. These demonstrated the feasibility of constructing membrane protein-mediated signal transduction systems in cell-free environments.

More complex networks and genetic circuits can achieve more precise and sophisticated regulation of expression, minimizing off-target accumulation. Independent sensing modules generate distinct signals that serve as multiple inputs to a Boolean logic gate circuit–comprising AND, OR, and NOT gates–which subsequently drives the production of therapeutic molecules (Fig. 4C). For example, CFVs equipped with T-cell receptors can distinguish between cancer cell types using an AND gate that detects two specific antigen signals, inspired by the engineered T-cell receptor"SynNotch"[111]. Furthermore, by integrating different extracellular and intracellular receptor domains, CFVs can generate multiple receptor orthologs, thereby broadening their range of recognizable molecules [112, 113]. Combining two or more logic gate circuits can increase the accuracy of biological therapeutic expression and reduce side effects. The input signals can be RNA or proteins, such as small transcription activator RNA and toehold switches [114, 115]. Further incorporation of a negative feedback system in which excess therapeutic molecules self-inhibit their own expression may prevent drug accumulation to the point of producing toxic effects. For example, synthetic closed-loop circuits that produce therapeutic molecules and their inhibitors can regulate the transcription and activity of therapeutics. Circuits have been biomedically applied to restore the homeostasis of target molecules [116–119]. Once these sophisticated gene expression mechanisms are established in CFPS systems, CFVs may serve as advanced platforms for efficient therapeutic production, surpassing conventional delivery vesicles.

### Evaluating the potential of CFV as a therapeutic delivery system

Beyond their programmability, CFVs offer several advantages as vesicle-based delivery systems. This is especially evident when compared to extracellular vesicles (EVs), which, despite their superior target specificity, face challenges in scalability and drug loading efficiency [120]. Notably, drug-loaded exosomes have demonstrated high therapeutic efficacy owing to their target specificity and are emerging as promising drug delivery vehicles [121–123].

CFVs enable streamlined development and rapid prototyping. Their composition and function can be optimized in vitro, enabling high-throughput functional testing without the complex steps required for cell line engineering. The rapid design‒test cycle and fine-tuning capabilities of CFVs can accelerate the development of high-quality delivery systems that support the controlled production and release of therapeutics [122].

Moreover, manufacturing CFVs is time and cost efficient. CFVs can autonomously produce and maintain therapeutic agents through specific signals until they rupture. This eliminates the need for separate purification of biological drugs from cells or media. Large-scale production is feasible through streamlined procedures, and various types of vesicles can be easily manufactured in large quantities. Both prokaryotic and eukaryotic CFPS systems have successfully demonstrated scalability [124–126], which is a critical advantage given the challenges of scaling EV-based therapeutics [127].

Finally, CFVs address the low encapsulation efficiency that limits EV applications [127]. Although advanced drug-loading methods for EVs have been developed, including chimeric exosome techniques and endogenous loading using parent cells [128–131], the efficiency of nucleic acid and protein loading typically remains below 50% [132]. However, CFVs spontaneously preserve the drug, and the produced biological drug is consistently captured within the vesicles, maintaining an encapsulation efficiency close to 100%. This eliminates the need for a separate drug-loading step, thereby enabling efficient production. Although some loss of the DNA expression cassette encoding biological drugs may occur during CFVs manufacturing, the associated costs are negligible compared to the potential loss of drug molecules in other systems. This cost-effectiveness, combined with high encapsulation efficiency and self-loading properties, makes CFVs a promising platform for therapeutic delivery.

### Applications of alternative vesicles in CFPS systems

In addition to liposomes, polymersomes, and microsomes, which are commonly used in CFPS systems, other types of vesicles have also shown promise. Dendrimersomes and hybrid vesicles require further investigation. Integrating these alternatives into CFPS can provide valuable insights into their effectiveness.

Dendrimersomes, which are formed from highly branched macromolecules known as dendrimers, have several advantages. First, these structures self-assemble into monodisperse vesicular nanoparticles, resulting in uniform particle sizes. Notably, dendrimers exhibit superior membrane permeability compared with liposomes, enabling more effective intracellular delivery of therapeutic agents [133]. Recent studies highlight their potential as safe carriers for various substances, including hydrophilic and amphiphilic MRI contrast agents [134]. These unique properties suggest that dendrimersomes could improve medical interventions by facilitating precise, targeted delivery of synthesized proteins to specific cells or tissues.

EV–liposome hybrid vesicles represent an innovative approach for drug delivery that combines the strengths of both components. EVs, which are naturally produced within cells, offer high biocompatibility and low toxicity. They excel in targeting specific cells or tissues through various intracellular signals, while protecting their cargo from external factors and enhancing drug efficacy via effective endosomal delivery. Key parameters in drug delivery systems include encapsulation efficiency (EE) — the percentage of drug enclosed within nanoparticles — and loading capacity (LC), which refers to the amount of drug per unit weight of the nanoparticle. Liposomes with modifiable structures complement EVs by providing high EE and LC and enable the transport of substantial quantities of cargo [135]. The modifiable structure of these materials enables customization during synthesis, enabling the implementation of customized properties and functions. This approach maintains the natural targeting characteristics of EVs, while incorporating the high EE, LC, and engineering flexibility of liposomes.

In conclusion, these alternative vesicles hold considerable potential for medical applications owing to their exceptional stability, efficiency, and functionality. When combined with CFPS systems acting as mobile factories, they enable precise production and targeted delivery of therapeutics, offering new possibilities for biomedical research and treatment development.

### Potential concerns requiring further investigation

Although CFVs achieve protein production at the milligram-per-liter level, their yields remain lower than those of cell-based protein production systems. This limitation in protein yield may hinder their efficacy as therapeutic agents. Insufficient protein content within vesicles necessitates the use of more vesicles to achieve therapeutic doses. However, injecting large quantities of vesicles may lead to increased toxicity. Therefore, optimizing protein expression within CFVs is crucial for achieving reliable therapeutic effects. Several strategies can enhance protein yields in CFPS systems, including optimizing cell extract preparation (e.g., refining cell lysis methods and performing prelysis reactions) and improving CFPS reaction conditions (e.g., screening proper energy regeneration sources and optimizing the concentrations of buffering agents, ions, monomers, and cofactors). In addition to these prerequisites, strategies to increase the productivity of CFPS systems must be explored. For example, developing a CFPS system in which the nucleic acid template and expressed proteins remain intact would be advantageous. In many cases, researchers have obtained extracts from engineered strains that lack or have reduced nuclease or protease activities, demonstrating enhanced protein productivity [136–142]. The introduction of protective sequences or inhibitors of degradative enzymes may also be effective. For instance, proteins such as GamS, Chi-site, single-chain Cro, and Ku increase transcriptional levels by preventing the degradation of the linear DNA template via the exonuclease activity of the RecBCD complex present in the *E. coli* cell-free extract [143, 144]. Similarly, DNA template methylation resulted in a 32% increase in the protein yield in *E. coli* cell-free extracts [144]. Despite these advancements, further improvements are needed to achieve gram-per-liter protein production for stable and robust synthesis. One approach is to engineer strains more precisely to enhance target protein production while minimizing unnecessary metabolic activity. Another is to reduce resource competition from endogenous gene expression, for example by using rare codons or incorporating orthogonal translation systems [145]. Additionally, optimizing energy regeneration systems—such as refining the ATP regeneration cycle to limit waste accumulation—represents another promising strategy.

A key aspect of CFVs-based therapeutic delivery is its reliance on genetic circuits or other regulatory mechanisms that function as programmed cells. Accurately predicting the behavior of designed genetic circuits in CFPS systems is essential. Researchers have developed artificial intelligence(AI)/machine learning(ML)-based mathematical models for CFPS systems to predict protein production and understand the limitations of these platforms. These models have identified numerous kinetic parameters in CFPS reactions and have been used to predict the behavior of genetic circuits such as bistable switches, coherent feed-forward loops, and CRISPR activation/interference circuits [146–148]. Microfluidic platforms have also been used to study genetic circuits in CFVs, enabling continuous supplement exchange and mimicking cellular environments with high predictive accuracy [146, 149–151]. It can be inferred that this approach has great potential for predicting the behavior of genetic circuits within CFVs. However, these studies were primarily conducted in vitro, and their relevance to in vivo conditions remains uncertain. Owing to the vast diversity of cell types, their abundance in the body, and the complexity of the surrounding microenvironment, numerous factors can influence the stability and chemical properties of synthetic membranes. These variations can affect the performance of CFPS systems and the operating conditions of genetic circuits. However, the factors contributing to discrepancies between the predicted outcomes and the actual behavior of CFVs in biological environments remain underexplored. Addressing these challenges requires further research, including the development of computational models and other analytical approaches to better predict and optimize CFVs functionality in vivo. By leveraging advanced computational tools—including programming environments like Python and MATLAB, and modeling platforms such as CellDesigner and COPASI. Furthermore, the incorporation of biofoundry platforms, which enable high-throughput automation of the design–build–test–learn cycle, is expected to accelerate the development of finely tuned AI/ML models for genetic circuit design and behavioral prediction. Through automated and iterative processes, biofoundries facilitate rapid data generation of large datasets and model development, thereby advancing the rational engineering and in vivo functionality of CFVs [152, 153]. Collectively, these advancements are expected to bridge the current gap between computational predictions and biological outcomes, thereby enhancing the potential of CFVs in therapeutic applications.

In the context of therapeutic delivery, it is imperative for CFVs to migrate toward the targeted regions and maintain prolonged durations [154, 155]. One promising strategy to enhance their targeting capability is the integration of specific peptides or receptor molecules onto the CFV surface. Notably, the incorporation of therapeutically relevant proteins into the vesicle membrane offers not only improved targeting but also functional benefits, such as facilitating direct biological activity at the site of delivery. Recently, Peruzzi et al. demonstrated that the application of the CFPS system can improve T cell activation and enhance the performance of nanoparticle-based mRNA delivery. This approach establishes a robust foundation for developing next-generation immunotherapeutics [60]. However, targeting efficiency remains suboptimal due to several limiting factors. One such limitation is the improper folding of membrane proteins, which can negatively impact their targeting efficacy. Moreover, synthetic vesicles are prone to recognition and elimination by various immune cells as they circulate in the bloodstream [155]. Several strategies—particularly liposomal formulations—have been developed to prevent rapid elimination after administration. For example, PEG conjugation has been shown to facilitate the ability of liposomes to evade clearance, whereas additional coating with functional proteins extends their presence in circulation [156, 157]. Incorporating ubiquitously expressed “self-marker” or its functional fragment has also been shown to reduce phagocytic uptake and prolong circulation time [158, 159]. However, the protein-coated vesicles may trigger immune responses owing to misfolding or membrane composition issue. EV-liposome hybrids offer a promising solution to this challenge. By integrating exosomal membranes into CFVs, their inherent homing ability can enhance targeting efficiency, whereas their native biological composition may help reduce immunogenicity [131, 160]. Exogenous proteins from cell extracts used in CFPS systems may be released upon vesicle rupture, potentially causing immunogenicity. Such limitations could potentially be overcome by utilizing cell-free extracts derived from the probiotic *E. coli* Nissle 1917, or, more broadly, from GRAS (Generally Recognized as Safe) microorganisms such as *Streptococcus thermophilus*, *Lactobacillus acidophilus*, and *Saccharomyces cerevisiae* [161]. Further validation and technological advancements in structural integrity, targeting efficacy, and immunogenicity are essential to facilitate the clinical application of CFVs.

## Conclusion

The integration of CFPS systems with vesicles represents an innovative approach for biomanufacturing and delivery of therapeutics. CFPS enables precise control over protein synthesis by allowing flexible environmental manipulation. Moreover, vesicles improve the stability, bioavailability, and targeted delivery of therapeutic agents. This synergistic combination expands applications beyond the production of valuable proteins to the development of advanced drug delivery systems.

By equipping CFVs with a “sensing and producing” program, therapeutic efficacy can be optimized through controlled drug release. One of the key challenges in drug delivery is non-specific targeting and unintended or excessive drug accumulation. To overcome these challenges, CFVs are designed to express therapeutic molecules only in response to specific signals, thereby ensuring precise drug release and minimizing off-target effects. Additionally, incorporating more complex genetic networks, such as Boolean logic gate systems and self-inhibitory mechanisms, provides further refinement in therapeutic control.

Despite these advantages, several challenges and unsolved questions remain, including the low efficiency of CFPS systems, accurate prediction of behavior of genetic circuits within CFVs in vivo, imperfect targeting capabilities, and potential immunogenicity of CFVs. Addressing these issues requires engineering the metabolic pathways of source microorganisms and developing improved energy regeneration and translation systems in CFPS. Biofoundry-assisted advances in AI and data-driven modeling are expected to enhance the design and predictability of genetic circuits for programmable biotherapeutic production. Targeting efficiency may be improved through membrane modifications such as PEGylation or conjugation with target-specific ligands. Additionally, using CFPS systems derived from host-compatible microbes or EV–liposome hybrids offer a promising strategy to reduce CFV-associated immune responses.

To advance CFVs as effective and clinically biosynthetic drug delivery systems, intensive research is required—ranging from optimizing vesicle composition and genetic circuit integration to validating CFV function in vivo. With continued innovation, CFVs hold immense potential as programmable drug carriers, capable of delivering tailored and responsive therapeutics. Such systems may pave the way for a new paradigm in precision medicine and revolutionize future healthcare.

## Data Availability

No datasets were generated or analysed during the current study.

## Abbreviations

CFPS: Cell-free protein synthesis
PTMs: Posttranslational modifications
CFVs: CFPS system-containing vesicles
GPCRs: G protein-coupled receptors
ER: Endoplasmic reticulum
scFvs: Single-chain variable fragments
IPTG: Isopropyl β-D-1-thiogalactopyranoside
ELP: Elastin-like polypeptide
CHO: Chinese hamster ovary
EVs: Extracellular vesicles
EE: Encapsulation efficiency
LC: Loading capacity

## References

[1] Yan X, Liu X, Zhao C, Chen G-Q. Applications of synthetic biology in medical and pharmaceutical fields. Signal Transduct Target Ther. 2023;8:199. 37169742 10.1038/s41392-023-01440-5 PMC10173249

[2] Dondapati SK, Stech M, Zemella A, Kubick S. Cell-free protein synthesis: a promising option for future drug development. BioDrugs. 2020;34:327–48. 32198631 10.1007/s40259-020-00417-y PMC7211207

[3] Powers J, Jang Y. Advancing biomimetic functions of synthetic cells through compartmentalized cell-free protein synthesis. Biomacromol. 2023;24:5539–50. 10.1021/acs.biomac.3c00879 37962115

[4] Smolskaya S, Logashina YA, Andreev YA. Escherichia coli extract-based cell-free expression system as an alternative for difficult-to-obtain protein biosynthesis. Int J Mol Sci. 2020;21:928. 32023820 10.3390/ijms21030928 PMC7037961

[5] Walker S, Busatto S, Pham A, Tian M, Suh A, Carson K, et al. Extracellular vesicle-based drug delivery systems for cancer treatment. Theranostics. 2019;9:8001–17. 31754377 10.7150/thno.37097 PMC6857056

[6] Sato W, Zajkowski T, Moser F, Adamala KP. Synthetic cells in biomedical applications. Wiley Interdiscip Rev Nanomed Nanobiotechnol. 2022;14:e1761. 34725945 10.1002/wnan.1761 PMC8918002

[7] Khambhati K, Bhattacharjee G, Gohil N, Braddick D, Kulkarni V, Singh V. Exploring the potential of cell-free protein synthesis for extending the abilities of biological systems. Front Bioeng Biotechnol. 2019;7:248. 31681738 10.3389/fbioe.2019.00248 PMC6797904

[8] Garenne D, Haines MC, Romantseva EF, Freemont P, Strychalski EA, Noireaux V. Cell-free gene expression. Nat Rev Methods Primers. 2021;1:49.

[9] Goh YH, Kim YC, Jeong SH, Joo S, Kwon YK, Yoon H, et al. Development of in vitro lycopene biosynthesis from geranyl pyrophosphate employing cell-free protein synthesis. Biotechnol Bioprocess Eng. 2024;29:661–72.

[10] Busso D, Kim R, Kim S-H. Using an Escherichia coli cell-free extract to screen for soluble expression of recombinant proteins. J Struct Funct Genomics. 2004;5:69–74. 15263845 10.1023/B:JSFG.0000029197.44728.c5

[11] Jin X, Hong SH. Cell-free protein synthesis for producing ‘difficult-to-express’ proteins. Biochem Eng J. 2018;138:156–64.

[12] Silverman AD, Karim AS, Jewett MC. Cell-free gene expression: an expanded repertoire of applications. Nat Rev Genet. 2020;21:151–70. 31780816 10.1038/s41576-019-0186-3

[13] Vilkhovoy M, Adhikari A, Vadhin S, Varner JD. The evolution of cell free biomanufacturing. Processes. 2020;8:675.

[14] Borkowski O, Koch M, Zettor A, Pandi A, Batista AC, Soudier P, et al. Large scale active-learning-guided exploration for in vitro protein production optimization. Nat Commun. 2020;11:1872. 32312991 10.1038/s41467-020-15798-5 PMC7170859

[15] Lu Y. Cell-free synthetic biology: engineering in an open world. Synthetic Syst Biotechnol. 2017;2:23–7. 10.1016/j.synbio.2017.02.003 PMC5625795 29062958

[16] Levine MZ, Gregorio NE, Jewett MC, Watts KR, Oza JP. Escherichia coli-Based Cell-Free Protein Synthesis: Protocols for a robust, flexible, and accessible platform technology. J Vis Exp. 2019. 10.3791/58882 30855561

[17] Focke PJ, Hein C, Hoffmann B, Matulef K, Bernhard F, Dötsch V, et al. Combining in vitro folding with cell free protein synthesis for membrane protein expression. Biochemistry. 2016;55:4212–9. 27384110 10.1021/acs.biochem.6b00488 PMC5021318

[18] Sullivan CJ, Pendleton ED, Sasmor HH, Hicks WL, Farnum JB, Muto M, et al. A cell-free expression and purification process for rapid production of protein biologics. Biotechnol J. 2016;11:238–48. 26427345 10.1002/biot.201500214

[19] Kim KJ, Lee SJ, Kim DM. Cell-free systems for a multi-pronged approach to next-generation therapeutics and diagnostics. Biotechnol Bioprocess Eng. 2024;29(2):233–9.

[20] Goerke AR, Swartz JR. Development of cell-free protein synthesis platforms for disulfide bonded proteins. Biotechnol Bioeng. 2008;99:351–67. 17626291 10.1002/bit.21567

[21] Ogonah OW, Polizzi KM, Bracewell DG. Cell free protein synthesis: a viable option for stratified medicines manufacturing? Curr Opin Chem Eng. 2017;18:77–83.

[22] Tinafar A, Jaenes K, Pardee K. Synthetic biology goes cell-free. BMC Biol. 2019;17:64. 31395057 10.1186/s12915-019-0685-x PMC6688370

[23] Pardee K, Slomovic S, Nguyen PQ, Lee JW, Donghia N, Burrill D, et al. Portable, on-demand biomolecular manufacturing. Cell. 2016;167:248-259.e12. 27662092 10.1016/j.cell.2016.09.013

[24] Hunt AC, Vögeli B, Hassan AO, Guerrero L, Kightlinger W, Yoesep DJ, et al. A rapid cell-free expression and screening platform for antibody discovery. Nat Commun. 2023;14:3897. 37400446 10.1038/s41467-023-38965-w PMC10318062

[25] Merk H, Gless C, Maertens B, Gerrits M, Stiege W. Cell-free synthesis of functional and endotoxin-free antibody Fab fragments by translocation into microsomes. Biotechniques. 2012;53:153–60. 22963477 10.2144/0000113904

[26] Jaroentomeechai T, Stark JC, Natarajan A, Glasscock CJ, Yates LE, Hsu KJ, et al. Single-pot glycoprotein biosynthesis using a cell-free transcription-translation system enriched with glycosylation machinery. Nat Commun. 2018;9:2686. 30002445 10.1038/s41467-018-05110-x PMC6043479

[27] Kightlinger W, Lin L, Rosztoczy M, Li W, DeLisa MP, Mrksich M, et al. Design of glycosylation sites by rapid synthesis and analysis of glycosyltransferases. Nat Chem Biol. 2018;14:627–35. 29736039 10.1038/s41589-018-0051-2

[28] Kightlinger W, Warfel KF, DeLisa MP, Jewett MC. Synthetic glycobiology: parts, systems, and applications. ACS Synth Biol. 2020;9:1534–62. 32526139 10.1021/acssynbio.0c00210 PMC7372563

[29] Sachse R, Dondapati SK, Fenz SF, Schmidt T, Kubick S. Membrane protein synthesis in cell-free systems: from bio-mimetic systems to bio-membranes. FEBS Lett. 2014;588:2774–81. 24931371 10.1016/j.febslet.2014.06.007

[30] Jacobs ML, Boyd MA, Kamat NP. Diblock copolymers enhance folding of a mechanosensitive membrane protein during cell-free expression. Proc Natl Acad Sci USA. 2019;116:4031–6. 30760590 10.1073/pnas.1814775116 PMC6410776

[31] Wuu JJ, Swartz JR. High yield cell-free production of integral membrane proteins without refolding or detergents. Biochim Biophys Acta. 2008;1778:1237–50. 18295592 10.1016/j.bbamem.2008.01.023

[32] Takeda H, Ogasawara T, Ozawa T, Muraguchi A, Jih P-J, Morishita R, et al. Production of monoclonal antibodies against GPCR using cell-free synthesized GPCR antigen and biotinylated liposome-based interaction assay. Sci Rep. 2015;5:11333. 26061673 10.1038/srep11333 PMC4462149

[33] Mayeux G, Gayet L, Liguori L, Odier M, Martin DK, Cortès S, et al. Cell-free expression of the outer membrane protein OprF of Pseudomonas aeruginosa for vaccine purposes. Life Sci Alliance. 2021;4:e202000958. 33972378 10.26508/lsa.202000958 PMC8127326

[34] Kalmbach R, Chizhov I, Schumacher MC, Friedrich T, Bamberg E, Engelhard M. Functional cell-free synthesis of a seven helix membrane protein: in situ insertion of bacteriorhodopsin into liposomes. J Mol Biol. 2007;371:639–48. 17586523 10.1016/j.jmb.2007.05.087

[35] Goren MA, Fox BG. Wheat germ cell-free translation, purification, and assembly of a functional human stearoyl-CoA desaturase complex. Protein Expr Purif. 2008;62:171–8. 18765284 10.1016/j.pep.2008.08.002 PMC2586813

[36] Gessesse B, Nagaike T, Nagata K, Shimizu Y, Ueda T. G-Protein coupled receptor protein synthesis on a lipid bilayer using a reconstituted cell-free protein synthesis system. Life (Basel). 2018;8:54. 30400226 10.3390/life8040054 PMC6316570

[37] Kaneda M, Nomura SM, Ichinose S, Kondo S, Nakahama K, Akiyoshi K, et al. Direct formation of proteo-liposomes by in vitro synthesis and cellular cytosolic delivery with connexin-expressing liposomes. Biomaterials. 2009;30:3971–7. 19423159 10.1016/j.biomaterials.2009.04.006

[38] Sevova ES, Goren MA, Schwartz KJ, Hsu F-F, Turk J, Fox BG, et al. Cell-free synthesis and functional characterization of sphingolipid synthases from parasitic trypanosomatid protozoa. J Biol Chem. 2010;285:20580–7. 20457606 10.1074/jbc.M110.127662 PMC2898309

[39] Kol M, Panatala R, Nordmann M, Swart L, van Suijlekom L, Cabukusta B, et al. Switching head group selectivity in mammalian sphingolipid biosynthesis by active-site-engineering of sphingomyelin synthases. J Lipid Res. 2017;58:962–73. 28336574 10.1194/jlr.M076133 PMC5408615

[40] Stech M, Merk H, Schenk JA, Stöcklein WFM, Wüstenhagen DA, Micheel B, et al. Production of functional antibody fragments in a vesicle-based eukaryotic cell-free translation system. J Biotechnol. 2012;164:220–31. 22982167 10.1016/j.jbiotec.2012.08.020

[41] Hershewe JM, Warfel KF, Iyer SM, Peruzzi JA, Sullivan CJ, Roth EW, et al. Improving cell-free glycoprotein synthesis by characterizing and enriching native membrane vesicles. Nat Commun. 2021;12:2363. 33888690 10.1038/s41467-021-22329-3 PMC8062659

[42] Ota S, Yoshizawa S, Takeuchi S. Microfluidic formation of monodisperse, cell-sized, and unilamellar vesicles. Angew Chem Int Ed. 2009;48:6533–7. 10.1002/anie.200902182 19644988

[43] Noireaux V, Libchaber A. A vesicle bioreactor as a step toward an artificial cell assembly. Proc Natl Acad Sci USA. 2004;101:17669–74. 15591347 10.1073/pnas.0408236101 PMC539773

[44] Kita H, Matsuura T, Sunami T, Hosoda K, Ichihashi N, Tsukada K, et al. Replication of genetic information with self-encoded replicase in liposomes. ChemBioChem. 2008;9:2403–10. 18785673 10.1002/cbic.200800360

[45] Lentini R, Martín NY, Forlin M, Belmonte L, Fontana J, Cornella M, et al. Two-way chemical communication between artificial and natural cells. ACS Cent Sci. 2017;3:117–23. 28280778 10.1021/acscentsci.6b00330 PMC5324081

[46] Caschera F, Lee JW, Ho KKY, Liu AP, Jewett MC. Cell-free compartmentalized protein synthesis inside double emulsion templated liposomes with in vitro synthesized and assembled ribosomes. Chem Commun. 2016;52:5467–9. 10.1039/c6cc00223d PMC4829377 27019994

[47] Nomura SM, Tsumoto K, Hamada T, Akiyoshi K, Nakatani Y, Yoshikawa K. Gene expression within cell-sized lipid vesicles. ChemBioChem. 2003;4:1172–5. 14613108 10.1002/cbic.200300630

[48] Peruzzi JA, Galvez NR, Kamat NP. Engineering transmembrane signal transduction in synthetic membranes using two-component systems. Proc Natl Acad Sci USA. 2023;120:e2218610120. 37126679 10.1073/pnas.2218610120 PMC10175788

[49] Saito H, Kato Y, Le Berre M, Yamada A, Inoue T, Yosikawa K, et al. Time-resolved tracking of a minimum gene expression system reconstituted in giant liposomes. ChemBioChem. 2009;10:1640–3. 19533718 10.1002/cbic.200900205

[50] Gonzales DT, Yandrapalli N, Robinson T, Zechner C, Tang TYD. Cell-free gene expression dynamics in synthetic cell populations. ACS Synth Biol. 2022;11:205–15. 35057626 10.1021/acssynbio.1c00376 PMC8787815

[51] Sunami T, Ichihashi N, Nishikawa T, Kazuta Y, Yomo T. Effect of liposome size on internal RNA replication coupled with replicase translation. ChemBioChem. 2016;17:1282–9. 27037959 10.1002/cbic.201500662

[52] Tang TYD, Cecchi D, Fracasso G, Accardi D, Coutable-Pennarun A, Mansy SS, et al. Gene-mediated chemical communication in synthetic protocell communities. ACS Synth Biol. 2018;7:339–46. 29091420 10.1021/acssynbio.7b00306

[53] Nishimura K, Matsuura T, Nishimura K, Sunami T, Suzuki H, Yomo T. Cell-free protein synthesis inside giant unilamellar vesicles analyzed by flow cytometry. Langmuir. 2012;28:8426–32. 22578080 10.1021/la3001703

[54] Niwa T, Sasaki Y, Uemura E, Nakamura S, Akiyama M, Ando M, et al. Comprehensive study of liposome-assisted synthesis of membrane proteins using a reconstituted cell-free translation system. Sci Rep. 2015;5:18025. 26667602 10.1038/srep18025 PMC4678891

[55] Kuruma Y, Stano P, Ueda T, Luisi PL. A synthetic biology approach to the construction of membrane proteins in semi-synthetic minimal cells. Biochim Biophys Acta. 2009;1788:567–74. 19027713 10.1016/j.bbamem.2008.10.017

[56] Kattan J, Doerr A, Dogterom M, Danelon C. Shaping liposomes by cell-free expressed bacterial microtubules. ACS Synth Biol. 2021;10:2447–55. 34585918 10.1021/acssynbio.1c00278 PMC8524656

[57] Ichihashi N, Matsuura T, Kita H, Hosoda K, Sunami T, Tsukada K, et al. Importance of translation-replication balance for efficient replication by the self-encoded replicase. ChemBioChem. 2008;9:3023–8. 19021140 10.1002/cbic.200800518

[58] Nourian Z, Roelofsen W, Danelon C. Triggered gene expression in fed-vesicle microreactors with a multifunctional membrane. Angew Chem Int Ed. 2012;51:3114–8. 10.1002/anie.201107123 22246637

[59] Hosoda K, Sunami T, Kazuta Y, Matsuura T, Suzuki H, Yomo T. Quantitative study of the structure of multilamellar giant liposomes as a container of protein synthesis reaction. Langmuir. 2008;24:13540–8. 18959434 10.1021/la802432f

[60] Peruzzi JA, Vu TQ, Gunnels TF, Kamat NP. Rapid generation of therapeutic nanoparticles using cell-free expression systems. Small Methods. 2023;7:e2201718. 37116099 10.1002/smtd.202201718 PMC10611898

[61] Steinkühler J, Peruzzi JA, Krüger A, Villaseñor CG, Jacobs ML, Jewett MC, et al. Improving cell-free expression of model membrane proteins by tuning ribosome cotranslational membrane association and nascent chain aggregation. ACS Synth Biol. 2024;13:129–40. 38150067 10.1021/acssynbio.3c00357

[62] Marques B, Liguori L, Paclet M-H, Villegas-Mendéz A, Rothe R, Morel F, et al. Liposome-mediated cellular delivery of active gp91(phox). PLoS ONE. 2007;2:e856. 17848987 10.1371/journal.pone.0000856 PMC1955831

[63] Lentini R, Santero SP, Chizzolini F, Cecchi D, Fontana J, Marchioretto M, et al. Integrating artificial with natural cells to translate chemical messages that direct E. coli behaviour. Nat Commun. 2014;5:4012. 24874202 10.1038/ncomms5012 PMC4050265

[64] Martino C, Kim S-H, Horsfall L, Abbaspourrad A, Rosser SJ, Cooper J, et al. Protein expression, aggregation, and triggered release from polymersomes as artificial cell-like structures. Angew Chem Int Ed. 2012;51:6416–20. 10.1002/anie.201201443 22644870

[65] Vogele K, Frank T, Gasser L, Goetzfried MA, Hackl MW, Sieber SA, et al. Towards synthetic cells using peptide-based reaction compartments. Nat Commun. 2018;9:3862. 30242152 10.1038/s41467-018-06379-8 PMC6154970

[66] Schreiber A, Huber MC, Schiller SM. Prebiotic protocell model based on dynamic protein membranes accommodating anabolic reactions. Langmuir. 2019;35:9593–610. 31287709 10.1021/acs.langmuir.9b00445

[67] Sharma B, Ma Y, Hiraki HL, Baker BM, Ferguson AL, Liu AP. Facile formation of giant elastin-like polypeptide vesicles as synthetic cells. Chem Commun. 2021;57:13202–5. 10.1039/d1cc05579h 34816831

[68] May S, Andreasson-Ochsner M, Fu Z, Low YX, Tan D, de Hoog HPM, et al. In vitro expressed GPCR inserted in polymersome membranes for ligand-binding studies. Angew Chem Int Ed. 2013;52:749–53. 10.1002/anie.201204645 23161746

[69] Nallani M, Andreasson-Ochsner M, Tan CWD, Sinner EK, Wisantoso Y, Geifman-Shochat S, et al. Proteopolymersomes: in vitro production of a membrane protein in polymersome membranes. Biointerphases. 2011;6:153–7. 22239807 10.1116/1.3644384

[70] Bernstein HD. Cotranslational translocation of proteins into canine rough microsomes. Curr Protoc Cell Biol. 2001;Chapter 11:Unit 11.4. 10.1002/0471143030.cb1104s00 18228309

[71] Stech M, Hust M, Schulze C, Dübel S, Kubick S. Cell-free eukaryotic systems for the production, engineering, and modification of scFv antibody fragments. Eng Life Sci. 2014;14:387–98. 25821419 10.1002/elsc.201400036 PMC4374706

[72] Stech M, Nikolaeva O, Thoring L, Stöcklein WFM, Wüstenhagen DA, Hust M, et al. Cell-free synthesis of functional antibodies using a coupled in vitro transcription-translation system based on CHO cell lysates. Sci Rep. 2017;7:12030. 28931913 10.1038/s41598-017-12364-w PMC5607253

[73] Sachse R, Wüstenhagen D, Šamalíková M, Gerrits M, Bier FF, Kubick S. Synthesis of membrane proteins in eukaryotic cell-free systems. Eng Life Sci. 2013;13:39–48.

[74] Dondapati SK, Kreir M, Quast RB, Wüstenhagen DA, Brüggemann A, Fertig N, et al. Membrane assembly of the functional KcsA potassium channel in a vesicle-based eukaryotic cell-free translation system. Biosens Bioelectron. 2014;59:174–83. 24727603 10.1016/j.bios.2014.03.004

[75] Zemella A, Grossmann S, Sachse R, Sonnabend A, Schaefer M, Kubick S. Qualifying a eukaryotic cell-free system for fluorescence based GPCR analyses. Sci Rep. 2017;7:3740. 28623260 10.1038/s41598-017-03955-8 PMC5473880

[76] Brödel AK, Sonnabend A, Roberts LO, Stech M, Wüstenhagen DA, Kubick S. IRES-mediated translation of membrane proteins and glycoproteins in eukaryotic cell-free systems. PLoS ONE. 2013;8:e82234. 24376523 10.1371/journal.pone.0082234 PMC3869664

[77] Quast RB, Sonnabend A, Stech M, Wüstenhagen DA, Kubick S. High-yield cell-free synthesis of human EGFR by IRES-mediated protein translation in a continuous exchange cell-free reaction format. Sci Rep. 2016;6:30399. 27456041 10.1038/srep30399 PMC4960648

[78] Gurramkonda C, Rao A, Borhani S, Pilli M, Deldari S, Ge X, et al. Improving the recombinant human erythropoietin glycosylation using microsome supplementation in CHO cell-free system. Biotechnol Bioeng. 2018;115:1253–64. 29384203 10.1002/bit.26554

[79] Thoring L, Wüstenhagen DA, Borowiak M, Stech M, Sonnabend A, Kubick S. Cell-free systems based on CHO cell lysates: optimization strategies, synthesis of “difficult-to-express” proteins and future perspectives. PLoS ONE. 2016;11:e0163670. 27684475 10.1371/journal.pone.0163670 PMC5042383

[80] Haueis L, Stech M, Kubick S. A cell-free expression pipeline for the generation and functional characterization of nanobodies. Front Bioeng Biotechnol. 2022;10:896763. 35573250 10.3389/fbioe.2022.896763 PMC9096027

[81] Nsairat H, Khater D, Sayed U, Odeh F, Al Bawab A, Alshaer W. Liposomes: structure, composition, types, and clinical applications. Heliyon. 2022;8:e09394. 35600452 10.1016/j.heliyon.2022.e09394 PMC9118483

[82] Berhanu S, Ueda T, Kuruma Y. Artificial photosynthetic cell producing energy for protein synthesis. Nat Commun. 2019;10:1325. 30902985 10.1038/s41467-019-09147-4 PMC6430821

[83] Lee KY, Park S-J, Lee KA, Kim S-H, Kim H, Meroz Y, et al. Photosynthetic artificial organelles sustain and control ATP-dependent reactions in a protocellular system. Nat Biotechnol. 2018;36:530–5. 29806849 10.1038/nbt.4140

[84] Moghimianavval H, Hsu Y-Y, Groaz A, Liu AP. In Vitro reconstitution platforms of mammalian cell-free expressed membrane proteins. Methods Mol Biol. 2022;2433:105–20. 34985740 10.1007/978-1-0716-1998-8_6 PMC8859695

[85] Ushiyama R, Koiwai K, Suzuki H. Plug-and-play microfluidic production of monodisperse giant unilamellar vesicles using droplet transfer across water-oil interface. Sens Actuators B Chem. 2022;355:131281.

[86] Filipczak N, Pan J, Yalamarty SSK, Torchilin VP. Recent advancements in liposome technology. Adv Drug Deliv Rev. 2020;156:4–22. 32593642 10.1016/j.addr.2020.06.022

[87] Saraf S, Jain A, Tiwari A, Verma A, Panda PK, Jain SK. Advances in liposomal drug delivery to cancer: an overview. J Drug Deliv Sci Technol. 2020;56:101549.

[88] Wagner A, Vorauer-Uhl K. Liposome technology for industrial purposes. J Drug Deliv. 2011;2011:591325. 21490754 10.1155/2011/591325 PMC3065896

[89] Buttitta G, Bonacorsi S, Barbarito C, Moliterno M, Pompei S, Saito G, et al. Scalable microfluidic method for tunable liposomal production by a design of experiment approach. Int J Pharm. 2024;662:124460. 39004291 10.1016/j.ijpharm.2024.124460

[90] Khadke S, Roces CB, Donaghey R, Giacobbo V, Su Y, Perrie Y. Scalable solvent-free production of liposomes. J Pharm Pharmacol. 2020;72:1328–40. 32671856 10.1111/jphp.13329

[91] Natsume Y, Wen HI, Zhu T, Itoh K, Sheng L, Kurihara K. Preparation of giant vesicles encapsulating microspheres by centrifugation of a water-in-oil emulsion. J Vis Exp. 2017;24(119):55282. 10.3791/55282 PMC5352288 28190062

[92] Adir O, Sharf-Pauker N, Chen G, Kaduri M, Krinsky N, Shainsky-Roitman J, et al. Preparing protein producing synthetic cells using cell free bacterial extracts, liposomes and emulsion transfer. J Vis Exp. 2020;158:e60829. 32391815 10.3791/60829 PMC7613214

[93] Sugiyama H, Osaki T, Takeuchi S, Toyota T. Perfusion chamber for observing a liposome-based cell model prepared by a water-in-oil emulsion transfer method. ACS Omega. 2020;5:19429–36. 32803036 10.1021/acsomega.0c01371 PMC7424586

[94] Yu JY, Chuesiang P, Shin GH, Park HJ. Post-processing techniques for the improvement of liposome stability. Pharmaceutics. 2021;13:1023. 34371715 10.3390/pharmaceutics13071023 PMC8309137

[95] Pasarin D, Ghizdareanu AI, Enascuta CE, Matei CB, Bilbie C, Paraschiv-Palada L, et al. Coating materials to increase the stability of liposomes. Polymers (Basel). 2023;15:782. 36772080 10.3390/polym15030782 PMC10004256

[96] Zhu H, Yang C, Ma K. Nanovesicles for transdermal drug delivery. In: Applications of nanovesicular drug delivery. Elsevier; 2022. p. 103–14.

[97] de Hoog H-PM, Lin JieRong EM, Banerjee S, Décaillot FM, Nallani M. Conformational antibody binding to a native, cell-free expressed GPCR in block copolymer membranes. PLoS ONE. 2014;9:e110847. 10.1371/journal.pone.0110847 PMC4203850 25329156

[98] Jang W-S, Park SC, Kim M, Doh J, Lee D, Hammer DA. The effect of stabilizer on the mechanical response of double-emulsion-templated polymersomes. Macromol Rapid Commun. 2015;36:378–84. 25515004 10.1002/marc.201400472

[99] Hasannia M, Lamei K, Abnous K, Taghdisi SM, Nekooei S, Nekooei N, et al. Targeted poly(L-glutamic acid)-based hybrid peptosomes co-loaded with doxorubicin and USPIONs as a theranostic platform for metastatic breast cancer. Nanomedicine. 2023;48:102645. 36549556 10.1016/j.nano.2022.102645

[100] Meng F, Zhong Z, Feijen J. Stimuli-responsive polymersomes for programmed drug delivery. Biomacromol. 2009;10:197–209. 10.1021/bm801127d 19123775

[101] Pagendarm HM, Stone PT, Baljon JJ, Aziz MH, Pastora LE, Wilson JT. Flash nanoprecipitation for the production of endosomolytic polymersomes for cytosolic drug delivery. 2021.

[102] Fonseca M, Jarak I, Victor F, Domingues C, Veiga F, Figueiras A. Polymersomes as the next attractive generation of drug delivery systems: definition, synthesis and applications. Materials (Basel). 2024;17:319. 38255485 10.3390/ma17020319 PMC10817611

[103] Fenz SF, Sachse R, Schmidt T, Kubick S. Cell-free synthesis of membrane proteins: tailored cell models out of microsomes. Biochim Biophys Acta. 2014;1838:1382–8. 24370776 10.1016/j.bbamem.2013.12.009

[104] Quast RB, Ballion B, Stech M, Sonnabend A, Varga BR, Wüstenhagen DA, et al. Cell-free synthesis of functional human epidermal growth factor receptor: Investigation of ligand-independent dimerization in Sf21 microsomal membranes using non-canonical amino acids. Sci Rep. 2016;6:34048. 27670253 10.1038/srep34048 PMC5037433

[105] Wilcock J, Ward L. Drug metabolism assessment: liver microsomes. In: The ADME encyclopedia: A comprehensive guide on biopharmacy and pharmacokinetics. Cham: Springer International Publishing; 2021. p. 1–9.

[106] Obeid MA, Al Qaraghuli MM, Alsaadi M, Alzahrani AR, Niwasabutra K, Ferro VA. Delivering natural products and biotherapeutics to improve drug efficacy. Ther Deliv. 2017;8:947–56. 29061102 10.4155/tde-2017-0060

[107] Said SS, Campbell S, Hoare T. Externally addressable smart drug delivery vehicles: current technologies and future directions. Chem Mater. 2019;31:4971–89.

[108] Adepu S, Ramakrishna S. Controlled drug delivery systems: current status and future directions. Molecules. 2021;26:5905. 34641447 10.3390/molecules26195905 PMC8512302

[109] Tenchov R, Bird R, Curtze AE, Zhou Q. Lipid Nanoparticles─from liposomes to mRNA vaccine delivery, a landscape of research diversity and advancement. ACS Nano. 2021;15:16982–7015. 34181394 10.1021/acsnano.1c04996

[110] Adamala KP, Martin-Alarcon DA, Guthrie-Honea KR, Boyden ES. Engineering genetic circuit interactions within and between synthetic minimal cells. Nat Chem. 2017;9:431–9. 28430194 10.1038/nchem.2644 PMC5407321

[111] Morsut L, Roybal KT, Xiong X, Gordley RM, Coyle SM, Thomson M, et al. Engineering customized cell sensing and response behaviors using synthetic notch receptors. Cell. 2016;164:780–91. 26830878 10.1016/j.cell.2016.01.012 PMC4752866

[112] Roybal KT, Williams JZ, Morsut L, Rupp LJ, Kolinko I, Choe JH, et al. Engineering T cells with customized therapeutic response programs using synthetic notch receptors. Cell. 2016;167:419-432.e16. 27693353 10.1016/j.cell.2016.09.011 PMC5072533

[113] Roybal KT, Rupp LJ, Morsut L, Walker WJ, McNally KA, Park JS, et al. Precision tumor recognition by T cells with combinatorial antigen-sensing circuits. Cell. 2016;164:770–9. 26830879 10.1016/j.cell.2016.01.011 PMC4752902

[114] Chappell J, Westbrook A, Verosloff M, Lucks JB. Computational design of small transcription activating RNAs for versatile and dynamic gene regulation. Nat Commun. 2017;8:1051. 29051490 10.1038/s41467-017-01082-6 PMC5648800

[115] Green AA, Silver PA, Collins JJ, Yin P. Toehold switches: de-novo-designed regulators of gene expression. Cell. 2014;159:925–39. 25417166 10.1016/j.cell.2014.10.002 PMC4265554

[116] Kemmer C, Gitzinger M, Daoud-El Baba M, Djonov V, Stelling J, Fussenegger M. Self-sufficient control of urate homeostasis in mice by a synthetic circuit. Nat Biotechnol. 2010;28:355–60. 20351688 10.1038/nbt.1617

[117] Rössger K, Charpin-El-Hamri G, Fussenegger M. A closed-loop synthetic gene circuit for the treatment of diet-induced obesity in mice. Nat Commun. 2013;4:2825. 24281397 10.1038/ncomms3825 PMC3868331

[118] Ausländer D, Ausländer S, Charpin-El Hamri G, Sedlmayer F, Müller M, Frey O, et al. A synthetic multifunctional mammalian pH sensor and CO2 transgene-control device. Mol Cell. 2014;55:397–408. 25018017 10.1016/j.molcel.2014.06.007

[119] Saxena P, Charpin-El Hamri G, Folcher M, Zulewski H, Fussenegger M. Synthetic gene network restoring endogenous pituitary-thyroid feedback control in experimental Graves’ disease. Proc Natl Acad Sci USA. 2016;113:1244–9. 26787873 10.1073/pnas.1514383113 PMC4747754

[120] Peng H, Ji W, Zhao R, Yang J, Lu Z, Li Y, et al. Exosome: a significant nano-scale drug delivery carrier. J Mater Chem B Mater Biol Med. 2020;8:7591–608. 32697267 10.1039/d0tb01499k

[121] Wang L, Yu X, Zhou J, Su C. Extracellular vesicles for drug delivery in cancer treatment. Biol Proced Online. 2023;25:28. 37946166 10.1186/s12575-023-00220-3 PMC10634104

[122] Ratajczak MZ, Ratajczak J. Extracellular microvesicles/exosomes: discovery, disbelief, acceptance, and the future? Leukemia. 2020;34:3126–35. 32929129 10.1038/s41375-020-01041-z PMC7685969

[123] Lee A, Lee JH, So C, Kim IG, Mok H. Exosome-immobilized porous microspheres for efficiently combined and prolonged cancer treatment. Biotechnol Bioprocess Eng. 2024;29:863–76.

[124] Voloshin AM, Swartz JR. Large-Scale Batch Reactions for Cell-Free Protein Synthesis. In: Spirin AS, Swartz JR, editors. Cell-Free Protein Synthesis. Weinheim, Germany: Wiley-VCH Verlag GmbH & Co. KGaA; 2007. p. 207–35.

[125] Katsura K, Matsuda T, Tomabechi Y, Yonemochi M, Hanada K, Ohsawa N, et al. A reproducible and scalable procedure for preparing bacterial extracts for cell-free protein synthesis. J Biochem. 2017;162:357–69. 28992119 10.1093/jb/mvx039 PMC7109869

[126] Gupta MD, Flaskamp Y, Roentgen R, Juergens H, Armero-Gimenez J, Albrecht F, et al. Scaling eukaryotic cell-free protein synthesis achieved with the versatile and high-yielding tobacco BY-2 cell lysate. Biotechnol Bioeng. 2023;120:2890–906. 37376851 10.1002/bit.28461

[127] Hussen BM, Faraj GSH, Rasul MF, Hidayat HJ, Salihi A, Baniahmad A, et al. Strategies to overcome the main challenges of the use of exosomes as drug carrier for cancer therapy. Cancer Cell Int. 2022;22:323. 36258195 10.1186/s12935-022-02743-3 PMC9580186

[128] Kwon S-H, Faruque HA, Kee H, Kim E, Park S. Exosome-based hybrid nanostructures for enhanced tumor targeting and hyperthermia therapy. Colloids Surf B Biointerfaces. 2021;205:111915. 34130212 10.1016/j.colsurfb.2021.111915

[129] Liu A, Yang G, Liu Y, Liu T. Research progress in membrane fusion-based hybrid exosomes for drug delivery systems. Front Bioeng Biotechnol. 2022;10:939441. 36051588 10.3389/fbioe.2022.939441 PMC9424752

[130] Lv Q, Cheng L, Lu Y, Zhang X, Wang Y, Deng J, et al. Thermosensitive exosome-liposome hybrid nanoparticle-mediated chemoimmunotherapy for improved treatment of metastatic peritoneal cancer. Adv Sci (Weinh). 2020;7:2000515. 32999828 10.1002/advs.202000515 PMC7509655

[131] Sato YT, Umezaki K, Sawada S, Mukai S, Sasaki Y, Harada N, et al. Engineering hybrid exosomes by membrane fusion with liposomes. Sci Rep. 2016;6:21933. 26911358 10.1038/srep21933 PMC4766490

[132] Zeng H, Guo S, Ren X, Wu Z, Liu S, Yao X. Current strategies for exosome cargo loading and targeting delivery. Cells. 2023;12:1416. 37408250 10.3390/cells12101416 PMC10216928

[133] Zhang D, Atochina-Vasserman EN, Maurya DS, Huang N, Xiao Q, Ona N, et al. One-component multifunctional sequence-defined ionizable amphiphilic janus dendrimer delivery systems for mRNA. J Am Chem Soc. 2021;143:12315–27. 34324336 10.1021/jacs.1c05813

[134] Filippi M, Patrucco D, Martinelli J, Botta M, Castro-Hartmann P, Tei L, et al. Novel stable dendrimersome formulation for safe bioimaging applications. Nanoscale. 2015;7:12943–54. 26167654 10.1039/c5nr02695d

[135] Ducrot C, Loiseau S, Wong C, Madec E, Volatron J, Piffoux M. Hybrid extracellular vesicles for drug delivery. Cancer Lett. 2023;558:216107. 36841417 10.1016/j.canlet.2023.216107

[136] Michel-Reydellet N, Woodrow K, Swartz J. Increasing PCR fragment stability and protein yields in a cell-free system with genetically modified Escherichia coli extracts. J Mol Microbiol Biotechnol. 2005;9:26–34. 16254443 10.1159/000088143

[137] Seki E, Matsuda N, Kigawa T. Multiple inhibitory factor removal from an Escherichia coli cell extract improves cell-free protein synthesis. J Biosci Bioeng. 2009;108:30–5. 19577188 10.1016/j.jbiosc.2009.02.011

[138] Seki E, Matsuda N, Yokoyama S, Kigawa T. Cell-free protein synthesis system from Escherichia coli cells cultured at decreased temperatures improves productivity by decreasing DNA template degradation. Anal Biochem. 2008;377:156–61. 18375196 10.1016/j.ab.2008.03.001

[139] Martin RW, Des Soye BJ, Kwon Y-C, Kay J, Davis RG, Thomas PM, et al. Cell-free protein synthesis from genomically recoded bacteria enables multisite incorporation of noncanonical amino acids. Nat Commun. 2018;9:1203. 29572528 10.1038/s41467-018-03469-5 PMC5865108

[140] Hong SH, Kwon Y-C, Martin RW, Des Soye BJ, de Paz AM, Swonger KN, et al. Improving cell-free protein synthesis through genome engineering of Escherichia coli lacking release factor 1. ChemBioChem. 2015;16:844–53. 25737329 10.1002/cbic.201402708 PMC4418657

[141] Schoborg JA, Hodgman CE, Anderson MJ, Jewett MC. Substrate replenishment and byproduct removal improve yeast cell-free protein synthesis. Biotechnol J. 2014;9:630–40. 24323955 10.1002/biot.201300383

[142] Hodgman CE, Jewett MC. Characterizing IGR IRES-mediated translation initiation for use in yeast cell-free protein synthesis. N Biotechnol. 2014;31:499–505. 25017988 10.1016/j.nbt.2014.07.001

[143] Yim SS, Johns NI, Noireaux V, Wang HH. Protecting linear DNA templates in cell-free expression systems from diverse bacteria. ACS Synth Biol. 2020;9:2851–5. 32926785 10.1021/acssynbio.0c00277

[144] Zhu B, Gan R, Cabezas MD, Kojima T, Nicol R, Jewett MC, et al. Increasing cell-free gene expression yields from linear templates in Escherichia coli and Vibrio natriegens extracts by using DNA-binding proteins. Biotechnol Bioeng. 2020;117:3849–57. 32816360 10.1002/bit.27538

[145] Kim J, Copeland CE, Seki K, Vögeli B, Kwon Y-C. Tuning the cell-free protein synthesis system for biomanufacturing of monomeric human Filaggrin. Front Bioeng Biotechnol. 2020;8:590341. 33195157 10.3389/fbioe.2020.590341 PMC7658397

[146] van Sluijs B, Maas RJM, van der Linden AJ, de Greef TFA, Huck WTS. A microfluidic optimal experimental design platform for forward design of cell-free genetic networks. Nat Commun. 2022;13:3626. 35750678 10.1038/s41467-022-31306-3 PMC9232554

[147] Pieters PA, Nathalia BL, van der Linden AJ, Yin P, Kim J, Huck WTS, et al. Cell-free characterization of coherent feed-forward loop-based synthetic genetic circuits. ACS Synth Biol. 2021;10:1406–16. 34061505 10.1021/acssynbio.1c00024 PMC8218305

[148] Tickman BI, Burbano DA, Chavali VP, Kiattisewee C, Fontana J, Khakimzhan A, et al. Multi-layer CRISPRa/i circuits for dynamic genetic programs in cell-free and bacterial systems. Cell Syst. 2022;13:215-229.e8. 34800362 10.1016/j.cels.2021.10.008

[149] van der Linden AJ, Yelleswarapu M, Pieters PA, Swank Z, Huck WTS, Maerkl SJ, et al. A Multilayer microfluidic platform for the conduction of prolonged cell-free gene expression. J Vis Exp. 2019;152:e59655. 10.3791/59655 31633684

[150] Gan R, Cabezas MD, Pan M, Zhang H, Hu G, Clark LG, et al. High-throughput regulatory part prototyping and analysis by cell-free protein synthesis and droplet microfluidics. ACS Synth Biol. 2022;11:2108–20. 35549070 10.1021/acssynbio.2c00050

[151] Karig DK, Jung S-Y, Srijanto B, Collier CP, Simpson ML. Probing cell-free gene expression noise in femtoliter volumes. ACS Synth Biol. 2013;2:497–505. 23688072 10.1021/sb400028c

[152] Zhang Q, Chen W, Qin M, Wang Y, Pu Z, Ding K, et al. Integrating protein language models and automatic biofoundry for enhanced protein evolution. Nat Commun. 2025;16:1553. 39934638 10.1038/s41467-025-56751-8 PMC11814318

[153] Kwon KK, Lee J, Kim H, Lee DH, Lee SG. Advancing high-throughput screening systems for synthetic biology and biofoundry. Curr Opinion Syst Biol. 2023;37:100487.

[154] Sawant RR, Torchilin VP. Challenges in development of targeted liposomal therapeutics. AAPS J. 2012;14:303–15. 22415612 10.1208/s12248-012-9330-0 PMC3326155

[155] Lu M, Zhao X, Xing H, Xun Z, Yang T, Cai C, et al. Liposome-chaperoned cell-free synthesis for the design of proteoliposomes: Implications for therapeutic delivery. Acta Biomater. 2018;76:1–20. 29625253 10.1016/j.actbio.2018.03.043

[156] Yokoe J, Sakuragi S, Yamamoto K, Teragaki T, Ogawara K, Higaki K, et al. Albumin-conjugated PEG liposome enhances tumor distribution of liposomal doxorubicin in rats. Int J Pharm. 2008;353:28–34. 18082345 10.1016/j.ijpharm.2007.11.008

[157] Watanabe M, Kawano K, Toma K, Hattori Y, Maitani Y. In vivo antitumor activity of camptothecin incorporated in liposomes formulated with an artificial lipid and human serum albumin. J Control Release. 2008;127:231–8. 18384903 10.1016/j.jconrel.2008.02.005

[158] Long KB, Beatty GL. Harnessing the antitumor potential of macrophages for cancer immunotherapy. Oncoimmunology. 2013;2:e26860. 24498559 10.4161/onci.26860 PMC3902119

[159] Rodriguez PL, Harada T, Christian DA, Pantano DA, Tsai RK, Discher DE. Minimal “Self” peptides that inhibit phagocytic clearance and enhance delivery of nanoparticles. Science. 2013;339:971–5. 23430657 10.1126/science.1229568 PMC3966479

[160] Zhang W, Ngo L, Tsao SCH, Liu D, Wang Y. Engineered cancer-derived small extracellular vesicle-liposome hybrid delivery system for targeted treatment of breast cancer. ACS Appl Mater Interfaces. 2023;15:16420–33. 36961985 10.1021/acsami.2c22749

[161] Ba F, Zhang Y, Ji X, Liu W-Q, Ling S, Li J. Expanding the toolbox of probiotic Escherichia coli Nissle 1917 for synthetic biology. Biotechnol J. 2024;19:e2300327. 37800393 10.1002/biot.202300327

